# Case report: Prenatal diagnosis of fetal non-compaction cardiomyopathy with bradycardia accompanied by *de novo CALM2* mutation

**DOI:** 10.3389/fped.2022.1012600

**Published:** 2022-11-23

**Authors:** Wen Zhang, Xiaohui Dai, Hanmin Liu, Lei Li, Shu Zhou, Qi Zhu, Jiao Chen

**Affiliations:** ^1^ Department of Ultrasonic Medicine, West China Second University Hospital of Sichuan University, Chengdu, China; ^2^ Key Laboratory of Birth Defects and Related Diseases of Women and Children, Ministry of Education, Sichuan University, Chengdu, China; ^3^ Department of Pediatrics, West China Second University Hospital of Sichuan University, Chengdu, China; ^4^ Department of Pathology, West China Second University Hospital of Sichuan University, Chengdu, China; ^5^ Department of Obstetrics, West China Second University Hospital of Sichuan University, Chengdu, China

**Keywords:** prenatal, ultrasound, non-compaction cardiomyopathy, bradycardia, CALM2 mutation

## Abstract

We herein report what appears to be the first case of fetal non-compaction cardiomyopathy in both ventricles accompanied by a mutation in the calmodulin gene (*CALM2*). A 25-year-old woman was referred to our hospital at 25^+1^ weeks of gestation for evaluation of fetal defects. Prenatal echocardiography showed biventricular non-compaction cardiomyopathy with sinus bradycardia. After termination of the pregnancy, fetal biventricular non-compaction cardiomyopathy was confirmed by autopsy and histopathologic examination. Additionally, whole-exome sequencing of genomic DNA demonstrated a *de novo* heterozygous mutation (c.389A > G; p.D130G) in *CALM2*, whereas the parents were normal. In this case report, we highlight the importance of prenatal ultrasound and genetic testing in fetal non-compaction cardiomyopathy with arrhythmia.

## Introduction

Non-compaction cardiomyopathy (NCCM) is a rare disorder that frequently manifests as monogenic diseases, especially neuromuscular disorders and chromosomal defects, and it was first reported on autopsy in 1969 (1). The incidence of NCCM in the general population ranges from 0.05% to 0.25%, whereas the incidence in children may reach 9.2% (2). NCCM is characterized by increased numbers of prominent trabeculations and deep intertrabecular spaces. Additionally, NCCM combined with arrhythmia has been rarely reported during the prenatal period. With the development of medical imaging techniques, the detection rate of NCCM has increased. Prenatal ultrasound is the primary and most convenient modality and can be used to recognize fetal arrhythmias. Thus, it is possible to identify NCCM with arrhythmia as early as the fetal period. As a rare genetic cardiomyopathy, NCCM is regulated by various genes that are involved in encoding ion channels, sarcomeres, and chaperone proteins. The related ion channel genes mainly include *SCN5A*, *RYR2*, *KCNQ1*, and *HCN4* (3). However, involvement of the calmodulin gene (*CALM2*) in fetal NCCM has been rarely reported. *CALM2* is a Ca^2+^-signaling gene that encodes for calmodulin, which is a multifunctional Ca^2+^-binding protein (4). Calmodulin is also an important calcium-sensitive signal transduction protein involved in regulating almost every cardiac ion channel through calcium/calmodulin-dependent protein kinase II (5, 6), and calmodulin may simultaneously contribute to cardiomyopathy and arrhythmia. We herein present the first case of fetal NCCM in both ventricles combined with sinus bradycardia and *CALM2* mutation at 25^+1^ weeks of gestation.

## Case description

**Figure 1:**
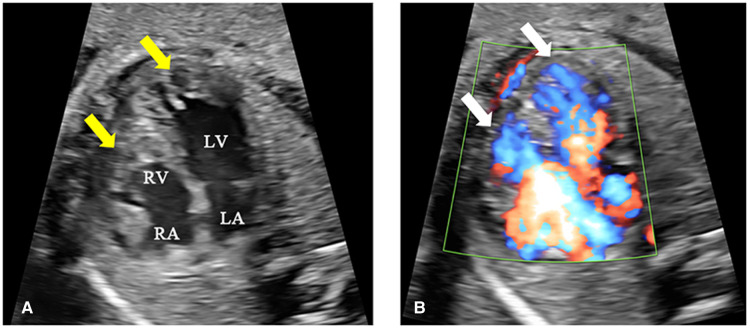
Fetal echocardiography at 25^+1^ weeks of gestation. (**A**) The two-dimensional ultrasound image shows increased numbers of prominent trabeculations and deep intertrabecular spaces in both ventricles (yellow arrow), especially at the left ventricular apex. The ratio of non-compaction (6 mm) to compaction (2 mm) was 3:1. (**B**) The color Doppler ultrasound image shows blood perfusing the intertrabecular recesses (white arrow). LA, left atrium; LV, left ventricle; RA, right atrium; RV, right ventricle.

**Figure 2:**
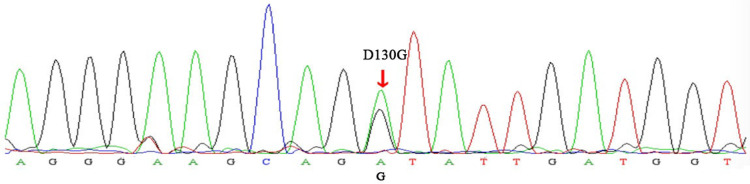
Sanger sequencing electropherogram. The variant (c.389A > G) demonstrated the replacement of a conserved aspartic acid residue at position 130 with glycine (p.D130G) in the *CALM2* gene (red arrow).

**Figure 3:**
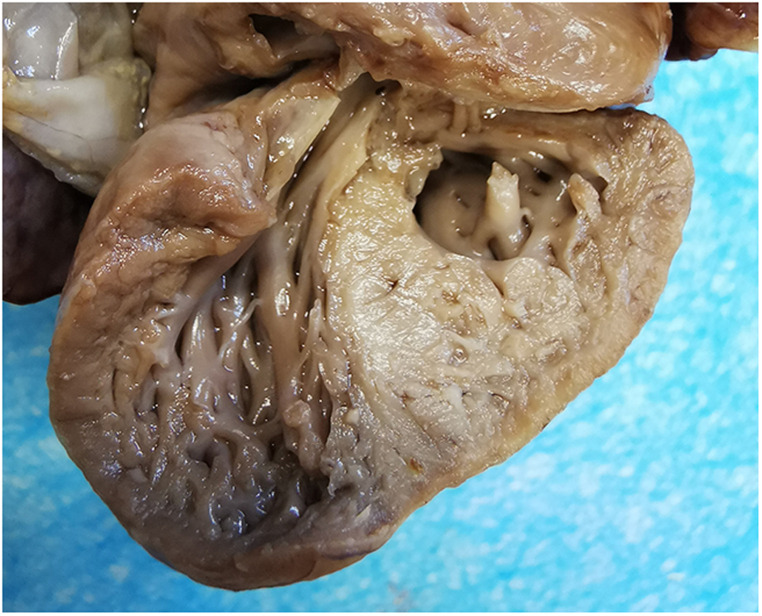
Dissected autopsy specimen. The specimen showed excessive trabeculae and deep intertrabecular recesses within the biventricular myocardium.

**Figure 4:**
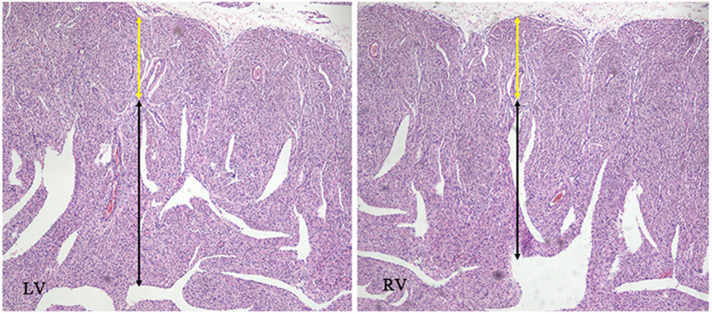
Histopathologic appearance of the myocardium at low magnification (hematoxylin and eosin, ×40). The images were compatible with non-compaction cardiomyopathy, with cardiomyocyte disarray in the non-compacted layer (black arrow) in opposition to regular cardiomyocytes in the compacted layer (yellow arrow).

A 25-year-old woman (gravida 1, para 0) was referred to our hospital at 25^+1^ weeks of gestation for evaluation of fetal defects. The patient was allergic to penicillin. Both parents were healthy, and there was no family history of birth defects or exposure to any specific teratogenic agents. A prenatal two-dimensional ultrasonographic investigation (3.0–5.0 MHz) (Voluson E10; GE Healthcare, Chicago, IL, United States) showed dilated ventricles (*Z*-score of left ventricular end-diastolic dimension: 2.51, Z-score of right ventricular end-diastolic dimension: 2.32), an increased cardiac area/thoracic area ratio (0.56), slight pericardial effusion, and extensive trabeculations in both ventricles. We found that in the left ventricle, the compacted layer became thinner (2 mm) and the non-compacted layer became thicker (6 mm), and in the right ventricle, the compacted layer became thinner (1.5 mm) and the intertrabecular space reached deeply into the epicardium. The ratio of non-compacted to compacted myocardium (N/C ratio) in the left and right ventricle was 3 and 2, respectively (Figure 1A). Color Doppler revealed blood perfusion to the intertrabecular recesses (Figure 1B). The heart rate was 101 bpm, and the atrioventricular (AV) interval was 133 ms. Therefore, the prenatal ultrasound diagnosis was biventricular NCCM with sinus bradycardia and pericardial effusion. Two weeks later, the fetal heart showed no significant improvement. The parents opted for pregnancy termination at 28 weeks' gestation after prenatal counseling, and heart autopsy and whole-exome sequencing (WES) were performed after obtaining the parents' informed consent. At autopsy, the biventricular wall contained increased numbers of prominent trabeculae and deep intertrabecular recesses (Figure 2). Histopathologic examination confirmed fetal NCCM (Figure 3). Genomic DNA was extracted from the muscle of the fetus to perform WES. The result demonstrated a *de novo* heterozygous mutation (c.389A > G; p. D130G) in *CALM2* (Figure 4). According to the current American College of Medical Genetics guidelines, the *CALM2* mutation was preliminarily determined to be the pathogenic variant (PS2 + PS4 + PM1 + PM2 + PM5 + PP3). The filtering cascades for the WES data of other variant genes are listed in Supplementary Table S1. The sequencing results of the parents were normal. The *CALM2* variant was not found in either the largest general population database (gnomAD, http://gnomad-sg.org) or the in-house control database.

## Discussion

NCCM is a rare cardiomyopathy with various genotypic and phenotypic manifestations. It is categorized as a primary genetic cardiomyopathy by the American Heart Association and as an unclassified cardiomyopathy by the European Society of Cardiology (7). The diagnosis of NCCM is complicated in fetal life, and there is no uniform standard. At present, many scholars diagnose fetal NCCM by reference to pediatric or adult criteria, mainly using the N/C ratio. According to a study by Stöllberger et al. (8), the diagnostic criteria for NCCM by echocardiography during pregnancy are as follows: at least four trabeculations protruding apically to the papillary muscle of the left ventricle visible in one imaging plane in end-diastole, a two-layered structure with epicardial compacted and endocardial noncompacted layers and an N/C ratio of ≥2, and perfusion of intraventricular blood into the intertrabecular spaces in color Doppler imaging. Fetal NCCM has its own specific imaging features. First, during development of the fetal cardiac structure, the N/C ratio of the myocardium in the normal fetus is much higher than that in a child or adult. Therefore, when the N/C ratio of the myocardium is about 2, we should be alert to the occurrence of NCCM and establish follow-up plans to observe the tendency of prominent trabeculations during the pregnancy. Second, because of the right ventricular dominance of the fetal circulation, fetal NCCM always involves both ventricles (9). By contrast, pediatric or adult NCCM most commonly occurs in the left ventricle; it rarely involves both ventricles, and isolated right ventricular NCCM is even rarer. Involvement of the right ventricle often implies a poor prognosis (10). In the published literature, most of the ultrasonic diagnostic criteria of right ventricular NCCM are based on the left ventricle; however, the right ventricle has more trabeculae, and its anatomical and morphological characteristics increase the difficulty of diagnosis of right ventricular NCCM. Fazio et al. (11) reported that the key to diagnosis of right ventricular NCCM is a significant increase of trabeculae in the right ventricle accompanied by dilation of this ventricle. The abnormal manifestations of the fetal right ventricle in the present case included a thin compacted layer, deep intertrabecular space, and dilated right ventricle. As noted by Fazio et al. (11), we consider that increased trabeculae within and enlargement of the right ventricle are the most important abnormalities for the diagnosis of fetal right ventricular NCCM and can provide instructive information for prenatal counseling.

NCCM in children and adults is always accompanied by arrhythmia. Kayvanpour et al. (12) found that the incidence of arrhythmias reached 61%, including conduction system diseases (26%), supraventricular tachycardia (17%), and sustained or non-sustained ventricular tachycardia (18%). Srivastava et al. (13) found that patients with NCCM had various electrocardiographic abnormalities, the most common of which were early repolarization and a prolonged QTc interval. Additionally, the types of arrhythmias were related to age. For example, Wolff-Parkinson-White syndrome and ventricular tachycardia were more common in children, and atrial fibrillation and other ventricular arrhythmias were more common in adults (13). However, prenatal diagnosis of fetal NCCM combined with arrhythmia has rarely been reported. We have herein presented the first case of fetal NCCM in both ventricles combined with sinus bradycardia. The normal fetal heart rate ranges from 120 to 160 bpm. Fetal bradyarrhythmia, which is defined as a heart rate of <110 bpm and mainly includes sinus bradycardia (16.9%) and AV block (38.2%) (14), is related to fetal hypoxia, abnormal heart structure, and maternal connective tissue disease. Sustained bradyarrhythmia can lead to cardiac function impairment manifesting as cardiac effusion in the fetus (15), as in the present case, suggesting a poor prognosis. Fetal echocardiography is the most commonly used method for diagnosing fetal arrhythmia. The AV interval is a key parameter for identifying the type of bradyarrhythmia. The normal fetal AV interval ranges from 112 to 130 ms (16). The AV interval of the fetus in this case was 133 ms; because it was <150 ms, it did not meet the diagnostic criteria for AV block (17). Therefore, the fetal arrhythmia type was considered to be sinus bradycardia. Sinus bradycardia is found in 40% of cases of fetal long QT syndrome (LQTS) during the prenatal examination (18). The fetal findings combined with the WES findings of the family in this case demonstrated a new mutation in *CALM2*. Therefore, we highly suspected that the fetus had NCCM combined with LQTS. Fetal magnetocardiography is currently the most consistent and reliable technique for diagnosis of LQTS because it can provide a fetal electrocardiographic-like signal to definitively demonstrate QTc prolongation (19). However, because this advanced device was unavailable in the present case, we were unable to prove the presence of QTc prolongation using prenatal echocardiography. Additionally, because the parents chose to induce labor, we were unable to definitively determine whether the fetus had LQTS.

NCCM can be familial or sporadic and may be isolated or accompanied by other cardiac diseases. The etiology of NCCM is complex and still unclear. Although at least 40 gene mutations are reportedly associated with NCCM [e.g., *MYH7* and *PRDM1*6 (20–22)], few case reports of *CALM2* mutation in fetal NCCM have been published. A previous study demonstrated strong or definitive evidence for a causal relationship between *CALM2* mutation and atypical LQTS phenotypes, including marked sinus bradycardia or atrioventricular block as well as QT prolongation in infancy or early childhood (23). Limpitikul et al. (24) demonstrated that the potential mechanism of *CALM2* mutation-induced LQTS is a disruption of Ca^2+^/calmodulin-dependent inactivation of L-type Ca^2+^ channels. Because the *CALM2* gene is involved in regulating ion channels, it may simultaneously contribute to cardiomyopathy and arrhythmia. Three published cases indicated that *CALM2* mutation might have contributed to LQTS accompanied by cardiomyopathy (one case of hypertrophic cardiomyopathy and two cases of left ventricular NCCM), indicating the variant positions in *CALM2* (c.396T > G; p.D132E, c.394G > C; p.D132H, and c395A > G; p.D132G) (25–27). Our case adds a report of a novel *CALM2* mutation (c.389A > G; p.D130G) in fetal NCCM combined with sinus bradycardia and detected by WES, providing more information regarding the relationship between the *CALM2* gene and fetal NCCM combined with arrhythmia. Considering our findings in combination with previously reported findings (25–27), we highly suspect that *CALM2* variants are simultaneous involved in cardiomyopathy and arrhythmia (especially LQTS). However, further research is required to confirm this hypothesis and elucidate the pathogenic mechanism.

In summary, prenatal ultrasound is very important to diagnose fetal NCCM. We should pay attention not only to abnormalities of myocardial morphology but also to the fetal heart rhythm. When prenatal ultrasound in the fetal period shows a dilated heart combined with increased trabeculae, especially in the right ventricle, fetal NCCM should be highly suspected. If the size of the heart and the N/C ratio progressively increase during ultrasound follow-up, genetic testing should be performed. Furthermore, in cases of fetal NCCM combined with arrhythmia, genetic testing is strongly recommended to provide more information for prenatal consulting and clinical application of precision medicine.

## Data Availability

The datasets presented in this study can be found in online repositories. The names of the repository/repositories and accession number(s) can be found in the article/**Supplementary Material**.

## References

[1] Finsterer J Stollberger C Towbin JA. Left ventricular noncompaction cardiomyopathy: cardiac, neuromuscular, and genetic factors. Nat Rev Cardiol. (2017) 14(4):224–37. 10.1038/nrcardio.2016.207 28079110

[2] Engberding R Stöllberger C Ong P Yelbuz TM Gerecke BJ Breithardt G. Isolated non-compaction cardiomyopathy. Dtsch Arztebl Int. (2010) 107(12):206–13. 10.3238/arztebl.2010.0206 20386670 PMC2853150

[3] Sun H Liu X Hao X Zhou X Wang J Han J Case report: biventricular noncompaction cardiomyopathy with pulmonary stenosis and bradycardia in a Fetus with KCNH2 mutation. Front Genet. (2022) 13:821226. 10.3389/fgene.2022.821226 35281812 PMC8908010

[4] Kato K Isbell HM Fressart V Denjoy I Debbiche A Itoh H Novel CALM3 variant causing calmodulinopathy with Variable expressivity in a 4-generation family. Circ Arrhythm Electrophysiol. (2022) 15(3):e010572. 10.1161/CIRCEP.121.010572 35225649

[5] Boczek NJ Gomez-Hurtado N Ye D Calvert ML Tester DJ Kryshtal D Spectrum and prevalence of CALM1-, CALM2-, and CALM3-encoded calmodulin variants in long QT syndrome and functional characterization of a novel long QT syndrome-associated calmodulin missense variant, E141G. Circ Cardiovasc Genet. (2016) 9(2):136–46. 10.1161/CIRCGENETICS.115.001323 26969752 PMC4907364

[6] Urrutia J Aguado A Muguruza-Montero A Núñez E Malo C Casis O Villarroel A. The crossroad of Ion channels and calmodulin in disease. Int J Mol Sci. (2019) 20(2):400. 10.3390/ijms20020400 30669290 PMC6359610

[7] Chebrolu LH Mehta AM Nanda NC. Noncompaction cardiomyopathy: the role of advanced multimodality imaging techniques in diagnosis and assessment. Echocardiography. (2017) 34(2):279–89. 10.1111/echo.13435 28058741

[8] Stollberger C Wegner C Finsterer J. Fetal ventricular hypertrabeculation/noncompaction: clinical presentation, genetics, associated cardiac and extracardiac abnormalities and outcome. Pediatr Cardiol. (2015) 36(7):1319–26. 10.1007/s00246-015-1200-y 26008764

[9] Sun H Hao X Wang X Zhou X Zhang Y Liu X Genetics and clinical features of noncompaction cardiomyopathy in the fetal population. Front Cardiovasc Med. (2020) 7:617561. 10.3389/fcvm.2020.617561 33553264 PMC7854697

[10] Nappi L Vasciaveo L Sorrentino F Scutiero G Iannone P Greco P. Fetal noncompaction cardiomyopathy and histologic diagnosis of spongy myocardium: case report and review of the literature. Rev Bras Ginecol Obstet. (2018) 40(11):722–5. 10.1055/s-0038-1673677 30308683 PMC10309442

[11] Fazio G Lunetta M Grassedonio E Gullotti A Ferro G Bacarella D Noncompaction of the right ventricle. Pediatr Cardiol. (2010) 31(4):576–8. 10.1007/s00246-010-9652-6 20155258

[12] Kayvanpour E Sedaghat-Hamedani F Gi WT Tugrul OF Amr A Haas J Clinical and genetic insights into non-compaction: a meta-analysis and systematic review on 7598 individuals. Clin Res Cardiol. (2019) 108(11):1297–308. 10.1007/s00392-019-01465-3 30980206

[13] Srivastava S Yavari M Al-Abcha A Banga S Abela G. Ventricular non-compaction review. Heart Fail Rev. (2021) 27(4):1063–76. 10.1007/s10741-021-10128-3 34232438

[14] Yuan SM. Fetal arrhythmias: diagnosis and treatment. J Matern Fetal Neonatal Med. (2020) 33(15):2671–8. 10.1080/14767058.2018.1555804 30879368

[15] Lingman G Lundstrom NR Marsal K. Clinical outcome and circulatory effects of fetal cardiac arrhythmia. Acta Paediatr Scand Suppl. (1986) 329:120–6. 10.1111/j.1651-2227.1986.tb10398.x 3473900

[16] Pan M Zhang MX Zhao BW Mao YK Peng XH Yang Y Reference ranges and Z-scores of atrioventricular and ventriculoatrial time intervals in Normal fetuses. Int J Cardiovasc Imaging. (2021) 37(8):2419–28. 10.1007/s10554-021-02217-z 33723733

[17] Wojakowski A Izbizky G Carcano ME Aiello H Marantz P Otaño L. Fetal Doppler mechanical PR interval: correlation with fetal heart rate,gestational age and fetal sex. Ultrasound Obstet Gynecol. (2009) 34(5):538–42. 10.1002/uog.7333 19731250

[18] Wacker-Gussmann A Strasburger JF Cuneo BF Wakai RT. Diagnosis and treatment of fetal arrhythmia. Am J Perinatol. (2014) 31(7):617–28. 10.1055/s-0034-1372430 24858320 PMC4073210

[19] Desai L Wakai R Tsao S Strasburger J Gotteiner N Patel A. Fetal diagnosis of KCNQ1-variant long QT syndrome using fetal echocardiography and magnetocardiography. Pacing Clin Electrophysiol. (2020) 43(4):430–3. 10.1111/pace.13900 32168391 PMC7166171

[20] Hoedemaekers YM Cohen-Overbeek TE Frohn-Mulder I ME Dooijes D Majoor-Krakauer DF. Prenatal ultrasound diagnosis of MYH7 non-compaction cardiomyopathy. Ultrasound Obstet Gynecol. (2013) 41(3):3369. 10.1002/uog.12279 22859017

[21] Nomura Y Momoi N Hirono K Hata Y Takasaki A Nishida N A novel MYH7 gene mutation in a fetus with left ventricular noncompaction. Can J Cardiol. (2015) 31(1):103.e1–3. 10.1016/j.cjca.2014.11.012 25547560

[22] Delplancq G Tarris G Vitobello A Nambot S Sorlin A Philippe C Cardiomyopathy due to PRDM16 mutation: first description of a fetal presentation, with possible modifier genes. Am J Med Genet C Semin Med Genet. (2020) 184(1):129–35. 10.1002/ajmg.c.31766 31965688

[23] Adler A Novelli V Amin AS Abiusi E Care M Nannenberg EA An international, multicentered, evidence-based reappraisal of genes reported to cause congenital long QT syndrome. Circulation. (2020) 141(6):418–28. 10.1161/CIRCULATIONAHA.119.043132 31983240 PMC7017940

[24] Limpitikul WB Dick IE Tester DJ Boczek NJ Limphong P Yang W A precision medicine approach to the rescue of function on malignant calmodulinopathic long-QT syndrome. Circ Res. (2017) 120(1):39–48. 10.1161/CIRCRESAHA.116.309283 27765793 PMC5516949

[25] Zahavich L Tarnopolsky M Yao R Mital S. Novel association of a De Novo CALM2 mutation with long QT syndrome and hypertrophic cardiomyopathy. Circ Genom Precis Med. (2018) 11(10):e002255. 10.1161/CIRCGEN.118.002255 30354306

[26] Pipilas DC Johnson CN Webster G Schlaepfer J Fellmann F Sekarski N Novel calmodulin mutations associated with congenital long QT syndrome affect calcium current in human cardiomyocytes. Heart Rhythm. (2016) 13(10):2012–9. 10.1016/j.hrthm.2016.06.038 27374306 PMC5035189

[27] Makita N Yagihara N Crotti L Johnson CN Beckmann BM Roh MS Novel calmodulin mutations associated with congenital arrhythmia susceptibility. Circ Cardiovasc Genet. (2014) 7(4):466–74. 10.1161/CIRCGENETICS.113.000459 24917665 PMC4140998

